# Neighborhood Food Environment and Birth Weight Outcomes in New York City

**DOI:** 10.1001/jamanetworkopen.2023.17952

**Published:** 2023-06-12

**Authors:** Eliza W. Kinsey, Elizabeth M. Widen, James W. Quinn, Mary Huynh, Gretchen Van Wye, Gina S. Lovasi, Kathryn M. Neckerman, Ellen C. Caniglia, Andrew G. Rundle

**Affiliations:** 1 Department of Family Medicine & Community Health, Perelman School of Medicine, University of Pennsylvania, Philadelphia; 2 Department of Nutritional Sciences and Population Research Center, University of Texas at Austin; 3 Department of Epidemiology, Mailman School of Public Health, Columbia University, New York, New York; 4 Bureau of Vital Statistics, New York City Department of Health and Mental Hygiene, New York; 5 Epidemiology and Biostatistics, Urban Health Collaborative, Dornsife School of Public Health, Drexel University, Philadelphia, Pennsylvania; 6 Columbia Population Research Center, Columbia University, New York, New York; 7 Department of Biostatistics, Epidemiology & Informatics, Perelman School of Medicine, University of Pennsylvania, Philadelphia

## Abstract

**Importance:**

Infants born with unhealthy birth weight are at greater risk for long-term health complications, but little is known about how neighborhood characteristics (eg, walkability, food environment) may affect birth weight outcomes.

**Objective:**

To assess whether neighborhood-level characteristics (poverty rate, food environment, and walkability) are associated with risk of unhealthy birth weight outcomes and to evaluate whether gestational weight gain mediated these associations.

**Design, Setting, and Participants:**

The population-based cross-sectional study included births in the 2015 vital statistics records from the New York City Department of Health and Mental Hygiene. Only singleton births and observations with complete birth weight and covariate data were included. Analyses were performed from November 2021 to March 2022.

**Exposures:**

Residential neighborhood-level characteristics, including poverty, food environment (healthy and unhealthy food retail establishments), and walkability (measured by both walkable destinations and a neighborhood walkability index combining walkability measures like street intersection and transit stop density). Neighborhood-level variables categorized into quartiles.

**Main Outcomes and Measures:**

The main outcomes were birth certificate birth weight measures including small for gestational age (SGA), large for gestational age (LGA), and sex-specific birth weight for gestational age z-score. Generalized linear mixed-effects models and hierarchical linear models estimated risk ratios for associations between density of neighborhood-level characteristics within a 1-km buffer of residential census block centroid and birth weight outcomes.

**Results:**

The study included 106 194 births in New York City. The mean (SD) age of pregnant individuals in the sample was 29.9 (6.1) years. Prevalence of SGA and LGA were 12.9% and 8.4%, respectively. Residence in the highest density quartile of healthy food retail establishments compared with the lowest quartile was associated with lower adjusted risk of SGA (with adjustment for individual covariates including gestational weight gain z-score: risk ratio [RR], 0.89; 95% CI 0.83-0.97). Higher neighborhood density of unhealthy food retail establishments was associated with higher adjusted risk of delivering an infant classified as SGA (fourth vs first quartile: RR, 1.12; 95% CI, 1.01-1.24). The RR for the association between density of unhealthy food retail establishments and risk of LGA was higher after adjustment for all covariates in each quartile compared with quartile 1 (second: RR, 1.12 [95% CI, 1.04-1.20]; third: RR, 1.18 [95% CI, 1.08-1.29]; fourth: RR, 1.16; [95% CI, 1.04-1.29]). There were no associations between neighborhood walkability and birth weight outcomes (SGA for fourth vs first quartile: RR, 1.01 [95% CI, 0.94-1.08]; LGA for fourth vs first quartile: RR, 1.06 [95% CI, 0.98-1.14]).

**Conclusions and Relevance:**

In this population-based cross-sectional study, healthfulness of neighborhood food environments was associated with risk of SGA and LGA. The findings support use of urban design and planning guidelines to improve food environments to support healthy pregnancies and birth weight.

## Key Points

**Question:**

Is the healthfulness of the neighborhood food environment associated with birth weight outcomes?

**Findings:**

In this population-based cross-sectional study of 106 194 births in New York City, higher neighborhood density of healthy food retail establishments was associated with a reduced risk of delivering an infant classified as small for gestational age, while higher neighborhood density of unhealthy food retail establishments was associated with higher risk of having an infant born either small or large for gestational age.

**Meaning:**

The findings suggest that urban design and planning guidelines to improve the healthfulness of neighborhood food environments are needed to promote healthy pregnancies and birth weights.

## Teaser

This cross-sectional study assesses the association of neighborhood-level characteristics (poverty rate, food environment, and walkability) with birth weight outcomes in New York City.

## Introduction

Both infants born small for gestational age (SGA) (defined as birth weight <10th percentile for gestational age at birth and sex) and large for gestational age (LGA) (birth weight >90th percentile for gestational age [GA] at birth and sex) are at higher risk for future health complications, including heart disease, high blood pressure, and obesity and related comorbidities.^1,2,3,4,5^

Prior studies^6,7,8^ have demonstrated an association of neighborhood socioeconomic status (SES) characteristics (eg, poverty rate, neighborhood deprivation) with risk of SGA, with most finding that living in a lower SES neighborhood was associated with a significantly higher risk of having an infant born SGA. Lower neighborhood SES has also been shown to be associated with infants born LGA^9,10,11^; however, research in this area is limited and some studies have shown no estimated association.^6^

While the literature^12,13,14,15,16,17,18,19^ assessing the association between neighborhood built environment features and birth weight outcomes is scant, there exists a robust body of literature demonstrating an association of neighborhood walkability and food environment with weight-related outcomes within the general population. Numerous studies^12,13,14,15,16^ have examined walkability features (eg, land use mix, density of public transit infrastructure, and intersection density) or multicomponent indices of walkability and have found an association between living in areas of higher walkability and lower body mass index (BMI) or risk of overweight or obesity. There is evidence supporting an association between healthier food environments and weight-related outcomes; living both in closer proximity to healthy food retail establishments and in an area with a higher density of healthy food retail establishments have been associated with lower BMI.^14,17,18,19^ However, findings on the association between the food environment and BMI or overweight and/or obesity have not been as robust or consistent as the walkability literature.

A previous study^20^ by our research team that used New York City (NYC) birth records from 2015 found that living in a neighborhood with a higher density of unhealthy food outlets was associated with lower odds of excessive gestational weight gain (GWG). However, higher density of unhealthy food outlets was associated with higher neighborhood walkability, which was also associated with lower odds of excessive GWG. Given the positive association between GWG and birth weight,^21,22^ we hypothesized that GWG would act as a mediating variable in analyses of associations between the density of unhealthy food outlets and birth weight outcomes. Therefore, to assess this possibility, analyses were conducted with and without adjustment for GWG, and formal mediation analyses were performed.

## Methods

This cross-sectional study was classified as nonhuman participant research by the Columbia University Irving Medical Center institutional review board and thus was considered exempt from review, with a waiver of informed consent. The study followed the Strengthening the Reporting of Observational Studies in Epidemiology (STROBE) reporting guideline.

### Data and Cohort Selection

This study included data from births reported in the 2015 vital statistics records from the NYC Department of Health and Mental Hygiene (DOHMH). The DOHMH collects annual data on all births in NYC using medical records and self-report from the birthing parent. Only singleton births and observations with a plausible prepregnancy BMI (defined as BMI of 12-70; calculated as weight in kilograms divided by height in meters squared) or child birth weight (defined as 500-6000 g) were included. Births with GA less than 22 weeks or greater than 42 weeks were excluded. Observations missing height or weight data for the pregnant individual either at the time of birth or before pregnancy were also excluded, as were observations missing primary exposure or outcome variables for neighborhood or birth weight.

### Measures

#### Neighborhood Variables

The residential address of the pregnant individual was geocoded to the corresponding 2010 census block. Using a common metric in the literature^23^ for defining residential neighborhood, a 1-km buffer was created around the geographic centroid of each census block. After removing areas of water, the land area of the radial buffers was characterized for sociodemographic and built environment characteristics.^24^ To preserve the confidentiality of the health records, the NYC DOHMH performed the linkage between census block of residence and neighborhood variables. The combined individual- and neighborhood-level data were then analyzed with neighborhood-level variables categorized into quartiles. Quartile cut points were established using the distribution of the neighborhood-level variables across all census blocks in NYC.

The locations of retail food establishments and walkable destinations were identified using the 2014 National Establishment Time Series data set, which provides a census of businesses and institutions in the US.^25,26^ Following methods developed for a prior study^14^ by our research team of neighborhood characteristics and obesity risk in NYC, we a priori grouped food retail establishments into healthy and unhealthy categories based on whether the types of food sold support a healthy weight and then calculated the density of healthy and unhealthy food establishments for each radial buffer. For example, we classified full-service supermarkets and fruit and vegetable markets as healthy and fast-food outlets, convenience stores, and candy stores as unhealthy. Categories were based on evidence in the literature^27,28,29,30,31^ reporting associations of different types of food retail establishments with energy density of foods sold, diet quality, and BMI. Prior work has shown that these food environment classifications are associated with residents’ BMI in NYC.^14,32,33^

Using National Establishment Time Series data, we also calculated density of walkable destinations, including businesses and municipal resources, for each radial buffer.^26,34^ The 2015 neighborhood walkability index (NWI) was calculated using residential density, street intersection density, land use mix for 5 types of land use (residential, office, retail, education, and entertainment), subway stop density, and the ratio of retail building floor area to retail land area.^35,36,37^ Each scale component was calculated for the land area inside each census block centroid’s 1-km radial buffer. The components were then z-score transformed and summed for each buffer. The proportion of residents whose income was below the federal poverty level (poverty rate) was calculated by applying tract-level US Census American Community Survey data (2012-2016) to the radial buffers using standard areal weighting interpolation methods.^38^ The data sets and methods used for calculating neighborhood exposure variables have been previously described in more detail.^14,20^

#### Birth Weight and GWG Measurements

Birth weight was measured in grams and GA in weeks. Birth weight for GA z-scores were calculated using a 2017 birth weight for GA reference for US singleton births.^5^ SGA and LGA were defined as less than the 10th percentile and greater than the 90th percentile of sex-specific birth weight for GA z-scores, respectively. Gestational weight gain was the difference between prepregnancy weight (kg) and weight at time of delivery (kg). The GWG for GA z-score was calculated using z-score charts specific to prepregnancy BMI groups.^39,40,41^

#### Covariates

The pregnant individual’s prepregnancy BMI (continuous) was calculated using weight (kg) and height (m^2^), which were extracted from the birth record in which these measures were primarily inputted from medical records (although a small number were likely self-reported). Other individual demographic variables were self-reported. The pregnant individual’s education level was a categorical variable ranging from less than high school to doctorate or professional degree. Age was included as a continuous variable. A dichotomous variable for nativity was defined as US born (including US territories) or born outside the US. Parity was defined as an ordinal variable using previous live births (1, 2, or ≥3). A dichotomous variable for smoking status in the 3 months before or during pregnancy was included. A 3-level categorical variable for insurance type was used as a proxy for income (private insurance, public insurance, and self-pay, other, or unknown). We included pregnant individuals’ race and ethnicity as a confounder not because we assumed there is a biological basis for difference in birth weight outcomes by race and ethnicity but because race and ethnicity may serve as a proxy for social factors (eg, racism) that influence birth weight and are not captured by other individual-level or neighborhood characteristics.^42^ Categories included Asian or Pacific Islander, Black non-Hispanic, Puerto Rican, White non-Hispanic, other Hispanic, other non-Hispanic, and non-Hispanic of 2 or more races (the latter 2 were combined as other or mixed race non-Hispanic. The groups included as “other” were not listed in the data set.

### Statistical Analysis

Statistical analyses were performed from November 2021 to March 2022 sing Stata, version 15.1 (StataCorp LLC).^43^ Univariable and multivariable logistic (SGA and LGA) and linear (birth weight for GA) regression analyses were used to analyze the association between GWG and birth weight outcomes. To assess the association between neighborhood-level variables and SGA compared with appropriate weight for GA (AGA), we used generalized linear mixed-effects models with a Poisson distribution and robust SEs.^44,45^ Likewise, in separate analyses, generalized linear mixed-effects models were used to analyze associations between neighborhood-level variables and LGA compared with AGA. The models included a random intercept for NYC community district, which represents the most local unit of government in NYC and typically reflects well-known neighborhood areas.^14^ Due to high collinearity between neighborhood food environment and walkability variables, 6 separate models were fit; the first 2 assessed neighborhood density of healthy and unhealthy food retail establishments and neighborhood poverty rate simultaneously as exposures for (1) SGA vs AGA and (2) LGA vs AGA, models 3 and 4 assessed NWI scores and neighborhood poverty rate for (3) SGA vs AGA and (4) LGA vs AGA, and models 5 and 6 assessed density of neighborhood walkable destinations and neighborhood poverty for (5) SGA vs AGA and (6) LGA vs AGA (eTable 1 in Supplement 1). Hierarchical linear models were used to assess the association between neighborhood-level variables and sex-specific birth weight for GA z-score. All models were adjusted for individual characteristics, including maternal age, race and ethnicity, educational level, nativity, smoking status, parity, insurance type, child sex, and prepregnancy BMI.

In addition, models were fit with GWG z-score under the hypothesis that it was acting as a competitive mediating variable.^46,47,48^ Specifically, higher density of unhealthy food outlets was negatively associated with GWG in previous studies, and GWG was positively associated with LGA.^20,21,22^ To test the role of GWG as a competitive mediating variable, we conducted a mediation analysis using Stata macro by VanderWeele^49^ to estimate the direct effect, indirect effect, and total effect of higher density of unhealthy food outlets on LGA (eTable 2 in Supplement 1). Given the observational and cross-sectional nature of this study, we will refer to these as direct and indirect associations going forward because they represent associations from which causal conclusions cannot be drawn. An additional sensitivity analysis was performed restricting analyses to full-term births. All models were evaluated for significance at 2-sided *P* < .05.

## Results

Of 110 744 births reported in the 2015 vital statistics records in NYC, 3735 were excluded because they were not singleton births, 6 because BMI did not fit the criterion, 61 because birth weight did not fit the criterion, 48 because GA did not fit the criterion, 687 because of missing height and weight data for the pregnant individual, and 13 because of missing primary exposure and outcome variables. The final sample included 106 194 births. The mean (SD) age of pregnant individuals in the sample was 29.9 (6.1) years, 59.1% were married, and 44.4% were nulliparous. Additional characteristics for the final analytic sample of 106 194 births are presented in Table 1. The mean (SD) birth weight for GA z-score was −0.14 (1.06), 12.9% of births were SGA, and 8.4% were LGA. As described in the Methods section, the neighborhood quartiles were defined based on the distribution of neighborhood variables across all NYC census blocks; however, the births in the data set were not evenly distributed across all quartiles. Pregnant individuals tended to live in areas of the city with higher poverty rates, higher food density, and greater walkability. For example, 7.2% and 9.9% of the pregnant individuals lived in the first quartile for unhealthy food density and walkability, respectively, while 48.2% and 38.7% lived in the highest unhealthy food density and walkability quartiles, respectively.

**Table 1.** Characteristics of the Analytic Sample From the 2015 Vital Statistics Records From the New York City Department of Health and Mental Hygiene

Characteristic	Participants^a^				
	Total (N = 106 194)	Unhealthy food density^b^			
		Quartile 1 (n = 7647)	Quartile 2 (n = 14 254)	Quartile 3 (n = 33 790)	Quartile 4 (n = 51 214)
Maternal age, mean (SD), y	29.9 (6.1)	30.5 (5.9)	29.8 (5.8)	29.7 (6.0)	30.0 (6.1)
Marital status					
Married	62 720 (59.1)	4911 (64.2)	7847 (55.1)	19 378 (58.6)	30 584 (59.7)
Unmarried	43 474 (40.9)	2736 (35.8)	6407 (44.9)	13 701 (41.4)	20 630 (40.3)
Educational level					
Some high school	20 686 (19.5)	713 (9.3)	2224 (15.6)	6400 (19.3)	11 349 (22.2)
High school graduate	24 342 (22.9)	1432 (18.7)	3064 (21.5)	7705 (23.3)	12 141 (23.7)
Some college	17 020 (16)	1519 (19.9)	2867 (20.1)	5552 (16.8)	7082 (13.8)
Associate's degree	7088 (6.7)	712 (9.3)	1264 (8.9)	2352 (7.1)	2760 (5.4)
Bachelor's degree	20 726 (19.5)	1919 (25.1)	2911 (20.4)	6356 (19.2)	9540 (18.6)
Master's degree	12 217 (11.5)	1082 (14.1)	1599 (11.2)	3659 (11.1)	5877 (11.5)
Doctorate or professional degree	3887 (3.7)	255 (3.3)	293 (2.1)	991 (3.0)	2348 (4.6)
Missing	228 (0.2)	15 (0.2)	32 (0.2)	64 (0.2)	117 (0.2)
Race and ethnicity					
Asian or Pacific Islander	18 154 (17.1)	1340 (17.5)	2633 (18.5)	6152 (18.6)	8029 (15.7)
Black non-Hispanic	20 723 (19.5)	2000 (26.2)	4333 (30.4)	6556 (19.8)	7834 (15.3)
Puerto Rican	6866 (6.5)	514 (6.7)	1010 (7.1)	2036 (6.2)	3306 (6.5)
White non-Hispanic	33 130 (31.2)	2768 (36.2)	3399 (23.8)	9798 (29.6)	17 165 (33.5)
Other Hispanic^c^	25 776 (24.3)	889 (11.6)	2638 (18.5)	8038 (24.3)	14 211 (27.7)
Other or mixed race non-Hispanic^c^^,^^d^	1516 (1.4)	135 (1.8)	238 (1.7)	490 (1.5)	653 (1.3)
Missing	29 (<0.1)	1 (<0.1)	3 (<0.1)	9 (<0.1)	16 (<0.1)
Nativity					
Born outside US	56 626 (53.3)	3563 (46.6)	7879 (55.3)	18 823 (56.9)	26 361 (51.5)
US born	49 545 (46.7)	4082 (53.4)	6372 (44.7)	14 250 (43.1)	24 841 (48.5)
Missing	23 (<0.1)	2 (<0.1)	3 (<0.1)	6 (<0.1)	12 (<0.1)
Prepregnancy BMI category^e^					
Underweight	6059 (5.7)	335 (4.4)	760 (5.3)	1866 (5.6)	3098 (6.0)
Normal	56 019 (52.8)	3893 (50.9)	6868 (48.2)	17 210 (52.0)	28 048 (54.8)
Overweight	25 852 (24.3)	1915 (25.0)	3781 (26.5)	8262 (25.0)	11 894 (23.2)
Obesity	18 264 (17.2)	1504 (19.7)	2845 (20.0)	5741 (17.4)	8174 (16.0)
IOM GWG category					
Inadequate	27 941 (26.3)	1679 (22.0)	3456 (24.2)	8741 (26.4)	14 065 (27.5)
Recommended	33 867 (31.9)	2240 (29.3)	4251 (29.8)	10 536 (31.9)	16 840 (32.9)
Excessive	44 386 (41.8)	3728 (48.8)	6547 (45.9)	13 802 (41.7)	20 309 (39.7)
Smoking status^f^					
Smoking	2111 (2.0)	258 (3.4)	349 (2.4)	609 (1.8)	895 (1.7)
Nonsmoking	104 014 (98.0)	7388 (96.6)	13 900 (97.5)	32 459 (98.1)	50 267 (98.2)
Missing	69 (0.1)	1 (<0.1)	5 (<0.1)	11 (<0.1)	52 (0.1)
Birth weight for GA category					
SGA	13 727 (12.9)	940 (12.3)	1951 (13.7)	4415 (13.3)	6421 (12.5)
AGA	83 541 (78.7)	6063 (79.3)	11 072 (77.7)	25 834 (78.1)	40 572 (79.2)
LGA	8926 (8.4)	644 (8.4)	1231 (8.6)	2830 (8.6)	4221 (8.2)
Insurance type					
Private	37 744 (35.5)	3745 (49.0)	5155 (36.2)	10 849 (32.8)	17 995 (35.1)
Public	66 869 (63.0)	3691 (48.3)	8777 (61.6)	21 740 (65.7)	32 661 (63.8)
Self-pay, other, or unknown	1581 (1.5)	211 (2.8)	322 (2.3)	490 (1.5)	558 (1.1)
Previous live births					
0	47 132 (44.4)	3324 (43.5)	6220 (43.6)	14 518 (43.9)	23 070 (45.0)
1	32 529 (30.6)	2621 (34.3)	4574 (32.1)	10 200 (30.8)	15 134 (29.6)
≥2	26 520 (25.0)	1702 (22.3)	3460 (24.3)	8361 (25.3)	13 010 (25.4)

Abbreviations: AGA, appropriate gestational age; BMI, body mass index (calculated as weight in kilograms divided by height in meters squared); GA, gestational age; GWG, gestational weight gain; IOM, Institute of Medicine; LGA, large for gestational age; SGA, small for gestational age.

^a^ Data are presented as number (percentage) of participants unless otherwise indicated.

^b^ Quartile cut points were established using the distribution of the neighborhood-level variables across all census blocks in New York City.

^c^ Groups included as “other” were not given in the data set.

^d^ The categories of other non-Hispanic and non-Hispanic of 2 or more races were combined from the birth certificate data.

^e^ Underweight was defined as a BMI less than 18.5; normal, 18.5 to less than 25.0; overweight, 25.0 to less than 30.0; and obesity, 30.0 or higher.

^f^ Cigarette smoking in the 3 months before or during pregnancy.

Table 2 shows unadjusted and adjusted outcomes of SGA and LGA (compared with AGA) and birth weight for GA z-score based on GWG z-score. Higher GWG z-score was associated with lower adjusted odds of SGA (odds ratio [OR], 0.77; 95% CI, 0.76-0.79) and higher adjusted odds of LGA (OR, 1.51; 95% CI, 1.47-1.54). Higher GWG z-score was also associated with higher birth weight for GA z-score (β, 0.18; 95% CI, 0.17-0.19).

**Table 2.** Association Between GWG z-Score and Birth Weight for GA z-Score

GWG z-score	Unadjusted	Adjusted^a^	Sensitivity analysis restricted to full-term births^a^
SGA, OR (95% CI)	0.78 (0.77-0.79)	0.77 (0.76-0.79)	0.76 (0.74-0.77)
LGA, OR (95% CI)	1.50 (1.46-1.54)	1.51 (1.47-1.54)	1.53 (1.49-1.57)
Birth weight for GA z-score, β (95% CI)	0.18 (0.18-0.19)	0.18 (0.17-0.19)	0.19 (0.18-0.19)

Abbreviations: GA, gestational age; GWG, gestational weight gain; LGA, large for gestational age; OR, odds ratio; SGA, small for gestational age.

^a^ Adjusted for maternal age, race and ethnicity, nativity, educational level, smoking status, parity, prepregnancy body mass index (continuous), child sex, and insurance type.

Table 3 presents risk ratios (RRs) for SGA and LGA for each quartile of poverty rate and neighborhood food environment. In the unadjusted and adjusted models, the highest density quartile of healthy food compared with the lowest quartile was associated with lower risk of SGA (with adjustment for individual covariates including GWG z-score: RR, 0.89; 95% CI, 0.83-0.97). Living in neighborhoods with a higher density of unhealthy food was associated with higher risk of delivering an infant classified as SGA (fourth vs first quartile: RR, 1.12; 95% CI, 1.01-1.24).

**Table 3.** Neighborhood Food Environment and Birth Weight Outcomes

Outcome	Risk ratio (95% CI)			
	Unadjusted	Adjusted^a^	Adjusted including GWG^b^	Restricted to full-term births^b^
**Small for gestational age**				
Births, No.	97 268	96 975	96 961	90 469
Poverty rate, quartile^c^				
1	1 [Reference]	1 [Reference]	1 [Reference]	1 [Reference]
2	0.94 (0.87-1.02)	0.92 (0.86-0.99)	0.93 (0.86-1.00)	0.91 (0.85-0.98)
3	1.01 (0.94-1.09)	0.98 (0.91-1.05)	0.97 (0.91-1.04)	0.97 (0.89-1.05)
4	1.04 (0.95-1.12)	1.00 (0.92-1.09)	1.00 (0.92-1.08)	1.00 (0.92-1.09)
Healthy food density, quartile^d^				
1	1 [Reference]	1 [Reference]	1 [Reference]	1 [Reference]
2	0.97 (0.91-1.03)	0.99 (0.93-1.05)	0.99 (0.93-1.05)	0.98 (0.91-1.06)
3	0.94 (0.87-1.01)	0.96 (0.90-1.02)	0.96 (0.90-1.02)	0.96 (0.88-1.04)
4	0.88 (0.80-0.96)	0.90 (0.83-0.97)	0.89 (0.83-0.97)	0.88 (0.81-0.97)
Unhealthy food density, quartile^e^				
1	1 [Reference]	1 [Reference]	1 [Reference]	1 [Reference]
2	1.12 (1.03-1.21)	1.10 (1.03-1.19)	1.10 (1.02-1.18)	1.10 (1.00-1.20)
3	1.16 (1.05-1.28)	1.15 (1.05-1.26)	1.14 (1.04-1.24)	1.15 (1.03-1.27)
4	1.16 (1.03-1.30)	1.13 (1.02-1.25)	1.12 (1.01-1.24)	1.12 (1.01-1.25)
**Large for gestational age**				
Births, No.	92 467	92 200	92 185	85 865
Poverty rate, quartile^c^				
1	1 [Reference]	1 [Reference]	1 [Reference]	1 [Reference]
2	0.99 (0.92-1.08)	0.99 (0.93-1.07)	1.00 (0.93-1.07)	1.00 (0.94-1.07)
3	1.00 (0.92-1.09)	1.00 (0.92-1.08)	0.99 (0.92-1.08)	1.00 (0.92-1.08)
4	0.96 (0.87-1.06)	0.95 (0.88-1.04)	0.95 (0.88-1.03)	0.95 (0.88-1.03)
Healthy food density, quartile^d^				
1	1 [Reference]	1 [Reference]	1 [Reference]	1 [Reference]
2	0.95 (0.86-1.04)	0.96 (0.87-1.05)	0.96 (0.87-1.05)	0.95 (0.86-1.05)
3	0.92 (0.84-1.01)	0.95 (0.87-1.03)	0.95 (0.87-1.03)	0.95 (0.88-1.03)
4	0.89 (0.80-0.98)	0.94 (0.86-1.03)	0.95 (0.86-1.03)	0.95 (0.87-1.04)
Unhealthy food density, quartile^e^				
1	1 [Reference]	1 [Reference]	1 [Reference]	1 [Reference]
2	1.07 (0.98-1.17)	1.09 (1.02-1.17)	1.12 (1.04-1.20)	1.13 (1.04-1.21)
3	1.11 (1.00-1.23)	1.12 (1.03-1.22)	1.18 (1.08-1.29)	1.17 (1.07-1.29)
4	1.09 (0.96-1.23)	1.10 (0.98-1.22)	1.16 (1.04-1.29)	1.16 (1.04-1.29)

Abbreviation: GWG, gestational weight gain.

^a^ Adjusted for maternal age, race and ethnicity, nativity, educational level, smoking status, parity, child sex, insurance type, and prepregnancy body mass index (continuous).

^b^ Adjusted for maternal age, race and ethnicity, nativity, educational level, smoking status, parity, child sex, insurance type, prepregnancy body mass index (continuous), and GWG z-score.

^c^ Poverty rate quartile cut points (quartile 1: ≤0.097; quartile 2: >0.097; quartile 3: >.0.151; quartile 4: ≥0.230).

^d^ Healthy food density quartile cut points (quartile 1: ≤0.644; quartile 2: >0.644; quartile 3: >1.592; quartile 4: ≥3.409).

^e^ Unhealthy food density quartile cut points (quartile 1: ≤10.310; quartile 2: >10.310; quartile 3: >24.531; quartile 4: ≥50.618).

In the first adjusted model, there was a positive association between the density of unhealthy food retail establishments and risk of LGA for the second quartile (RR, 1.09; 95% CI, 1.02-1.17) and third quartile (RR, 1.12; 95% CI, 1.03-1.22) compared with the first quartile, but there was no association for the fourth quartile compared with the first. After adjustment for all covariates including GWG z-score, the RRs for the association of higher density of unhealthy food retail establishments with LGA was higher for each quartile (second: RR, 1.12 [95% CI, 1.04-1.20]; third: RR, 1.18 [95% CI, 1.08-1.29]; fourth: RR, 1.16; [95% CI, 1.04-1.29]). The increase in the estimated effect size was consistent with GWG acting as a competitive mediating variable in the data. The formal mediation analysis examining GWG as a mediating factor between unhealthy food density and LGA (eTable 2 in Supplement 1) found a significant indirect association (through higher GWG) for each of the upper 3 quartiles compared with the first (second: RR, 0.98 [95% CI, 0.97-0.99]; third: RR, 0.97 [95% CI, 0.95-0.98]; fourth: RR, 0.94 [95% CI, 0.93-0.96]). There were direct associations in the opposite direction for the second (RR, 1.16; 95% CI, 1.02-1.31) and third (RR, 1.17; 95% CI, 1.00-1.36) quartiles.

The risk of SGA was only associated with increased neighborhood poverty rate for the second quartile compared with the first, and no significant association was found between neighborhood poverty and LGA or birth weight for GA z-score. Likewise, there were no associations between healthy or unhealthy food density and birth weight for GA z-score or between NWI or walkable destinations and SGA, LGA, or birth weight for GA z-score (Table 4 and eTable 1 in Supplement 1). When restricted to term births, the findings for neighborhood characteristics and SGA and LGA were similar.

**Table 4.** Neighborhood Walkability Index and Birth Weight Outcomes

Outcome	Risk ratio (95% CI)			
	Unadjusted	Adjusted^a^	Adjusted including GWG^b^	Restricted to full-term births^b^
**Small for gestational age**				
Births, No.	97 243	96 950	96 936	90 445
Poverty rate, quartile^c^				
1	1 [Reference]	1 [Reference]	1 [Reference]	1 [Reference]
2	0.95 (0.87-1.04)	0.93 (0.86-1.01)	0.94 (0.87-1.01)	0.92 (0.85-1.00)
3	1.03 (0.97-1.11)	1.00 (0.93-1.07)	0.99 (0.93-1.06)	0.99 (0.92-1.06)
4	1.06 (0.98-1.14)	1.03 (0.95-1.11)	1.02 (0.95-1.10)	1.02 (0.94-1.10)
Neighborhood walkability index, quartile^d^				
1	1 [Reference]	1 [Reference]	1 [Reference]	
2	1.02 (0.95-1.09)	1.03 (0.96-1.10)	1.03 (0.96-1.09)	1.03 (0.97-1.10)
3	1.01 (0.94-1.09)	1.01 (0.94-1.08)	1.00 (0.94-1.07)	1.01 (0.94-1.07)
4	1.01 (0.94-1.09)	1.02 (0.95-1.09)	1.01 (0.94-1.08)	1.01 (0.94-1.08)
**Large for gestational age**				
Births, No.	92 438	92 171	92 156	85 837
Poverty rate, quartile^c^				
1	1 [Reference]	1 [Reference]	1 [Reference]	1 [Reference]
2	1.01 (0.93-1.09)	1.01 (0.94-1.08)	1.01 (0.95-1.08)	1.02 (0.96-1.08)
3	1.03 (0.94-1.12)	1.02 (0.94-1.11)	1.02 (0.95-1.10)	1.03 (0.96-1.11)
4	0.98 (0.89-1.08)	0.97 (0.90-1.05)	0.98 (0.91-1.05)	0.98 (0.92-1.05)
Neighborhood walkability index, quartile^d^				
1	1 [Reference]	1 [Reference]	1 [Reference]	1 [Reference]
2	0.99 (0.92-1.07)	1.00 (0.93-1.07)	1.03 (0.96-1.10)	1.03 (0.96-1.10)
3	0.97 (0.90-1.05)	1.01 (0.94-1.08)	1.04 (0.97-1.12)	1.05 (0.97-1.13)
4	0.97 (0.89-1.06)	1.01 (0.94-1.09)	1.06 (0.98-1.14)	1.06 (0.98-1.15)

Abbreviation: GWG, gestational weight gain.

^a^ Adjusted for maternal age, race and ethnicity, nativity, educational level, smoking status, parity, child sex, insurance type, and prepregnancy body mass index (continuous).

^b^ Adjusted for maternal age, race and ethnicity, nativity, educational level, smoking status, parity, child sex, insurance type, prepregnancy body mass index (continuous), and GWG z-score.

^c^ Poverty rate quartile cut points (quartile 1: ≤0.097; quartile 2: >0.097; quartile 3: >.0.151; quartile 4: ≥0.230).

^d^ Neighborhood walkability index quartile cut points (quartile 1: ≤−1.632; quartile 2: >−1.632; quartile 3: >−0.279; quartile 4: ≥1.067).

## Discussion

In this cross-sectional study, the risk of having an infant who was SGA was lower for pregnant individuals living in the highest density quartile of healthy food retail establishments, while an increasing density of unhealthy food retail establishments was associated with higher SGA risk. This association remained even after adjusting for GWG z-score, suggesting an association between healthfulness of the neighborhood food environment and healthy birth weight that was not solely influenced by weight gain during pregnancy. Living closer to healthy food retail establishments has been shown in some prior studies^14,18,50^ to be associated with a healthier diet (eg, higher consumption of fruit and vegetables), as has living in a neighborhood with greater density of healthy food retail outlets. Furthermore, numerous studies^51,52^ have shown that preconception and prenatal diets that are rich in fruits, vegetables, whole grains, legumes, and dairy are associated with reduced risk of adverse birth outcomes, including preterm birth and SGA.

The present analyses found that an increase in GWG by 1 SD was associated with 23% lower odds of SGA, 51% higher odds of LGA, and a 0.18-SD increase in birth weight for GA. These findings align with previous literature^21,22^ showing that GWG above the Institute of Medicine recommendations is associated with lower odds of SGA and higher odds of LGA and that, inversely, GWG below Institute of Medicine recommendations is associated with lower odds of LGA and higher odds of SGA.

Of relevance to the analyses of LGA presented herein, prior work^20^ by our research team found that higher density of unhealthy food outlets was associated with lower odds of excessive GWG. Given the high correlation between neighborhood walkability and unhealthy food density in NYC, we previously hypothesized that higher density of unhealthy food would be a proxy in our analyses for neighborhood walkability, which was associated with lower odds of excessive GWG.^20^ Unhealthy food outlets are commonly found near transit stops and in areas with higher population density and higher land use mix, which are all key components of neighborhood walkability. The previous findings,^20^ combined with results in the current study showing that higher density of unhealthy food outlets was associated with a higher risk of LGA only after adjusting for GWG z-score, suggests that GWG may have a competitive mediating effect in the unadjusted analyses of neighborhood food environment and LGA.^46^ That is, in the present analyses, higher density of unhealthy food outlets was associated with LGA through 2 pathways: one negatively associated with LGA through GWG and the other positively associated with LGA (Figure). In the presence of a competitive mediating effect, when one of the pathways is statistically blocked, which occurred in this analysis by controlling for GWG, the other pathway becomes apparent as a higher estimate for the association between the exposure and the outcome. The results of the formal mediation analysis showed that GWG was acting as a mediating factor, as there were direct and indirect associations but in different directions, which represents competitive mediation or a suppressor effect.^48^

**Figure.**
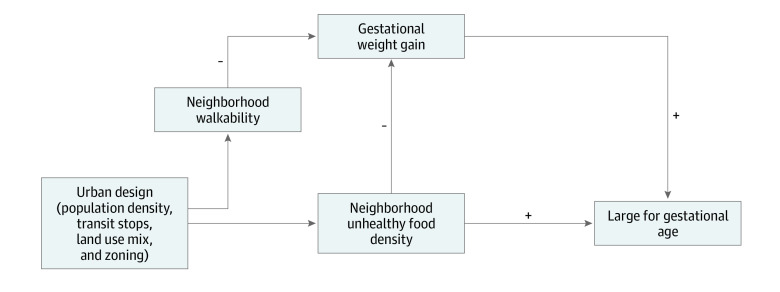
Conceptual Illustration of the Hypothesized Competitive Mediating Effect of Gestational Weight Gain in the Association Between Neighborhood Unhealthy Food Density and Large for Gestational Age Neighborhood walkability and unhealthy food density were highly correlated because they are both related to the antecedent urban design features shown in the figure.

Unlike previous studies^6,7,8^ showing an association between neighborhood SES and birth weight outcomes, we found no association between increasing neighborhood poverty rate quartiles and SGA, LGA, or birth weight for GA. While several studies^9,10,11^ have found a positive association between neighborhood SES or neighborhood deprivation and LGA, the research is limited and has shown both substantial mediation through prepregnancy BMI and differential outcomes by race. Furthermore, some studies^6^ have found a negative association between neighborhood SES and LGA. The literature^6,7,8,11^ demonstrating a positive association between neighborhood SES and risk of SGA is more substantial, making the null results in our analyses more surprising. Several possible explanations include our use of neighborhood poverty rate rather than a neighborhood deprivation index and the protective effects of immigrant enclaves, which could potentially offset the adverse health impacts of living in high-poverty neighborhoods.^53^

Contrary to a previous study^20^ by our research team that demonstrated a significant association between higher neighborhood walkability and reduced odds of excessive GWG, in the present analyses, walkability was not associated with birth weight outcomes. This finding may be attributed to several possible explanations. First, greater neighborhood walkability and resulting pedestrian activity may be sufficient structural and behavioral differences to improve GWG but may not be enough to alter risk of infant birth weight. Second, birth weight for GA, like BMI, is a rough measure of body composition, and it is possible that more refined infant adiposity data would elucidate a different association between walkability and infant weight outcomes.^54^

### Limitations

The study has several limitations, which have been previously reported in more detail.^20^ First, while medical records and anthropometric measurements from a clinical setting are believed to have been used for the majority of the height and weight data, some data in the sample may have been derived from self-report. However, self-reported errors in height and weight data are unlikely to alter the conclusions of the analyses, as previous research demonstrated that self-reported and measured anthropometric data produced similar estimates in health effects studies.^55^

The unique built environment characteristics of NYC (high density and low car ownership)^56^ may limit the generalizability of the findings to populations in different geographic contexts, particularly suburban and rural areas. Given that the percentage of the world's population living in urban areas is projected to grow to 68% by 2050, findings from urban settings such as NYC are relevant for much of the global population.^57^

The walkability measures used in the present study are based solely on urban form and planning concepts and do not include more experiential characteristics of the built environment such as aesthetic qualities or perceived safety.^36^ However, many of these experiential qualities correlate with neighborhood socioeconomic conditions, which was captured by adjustment in the analyses for neighborhood poverty rate.^36^ Lastly, opportunities for causal inference are limited by the observational and cross-sectional nature of the study.

## Conclusions

In this cross-sectional study, higher neighborhood density of healthy food retail establishments was associated with a reduced risk of delivering an infant classified as SGA, while higher neighborhood density of unhealthy food retail establishments was associated with higher risk of having an infant born either SGA or LGA. This research provides further evidence of a role of neighborhood environment features in pregnancy and birth outcomes. Well-established guidelines exist for improving perinatal health through diet and physical activity.^58,59^ However, due to limited research on the implications of active design for health during pregnancy, few wellness-oriented urban design and planning guidelines explicitly consider pregnant individuals and their infants. Given the long-lasting benefits of healthy pregnancies for parental and child health, this research provides further impetus for the use of urban design to support healthy weight and reduce the risk of unhealthy birth weight. Supporting the health of young children should be factored into cost-benefit analyses of built environment interventions to improve healthy food access across neighborhoods.

## Notes

Supplement 1. **eTable 1.** Walkable Destinations and Birth Weight Outcomes **eTable 2.** GWG Mediation Analysis for Unhealthy Neighborhood Food Environment and LGA

Supplement 2.
Data Sharing Statement
