# Supplementary material for: Neighborhood Food Environment and Birth Weight Outcomes in New York City
Source: JAMA Netw Open. 2023 Jun 12;6(6):e2317952. doi: 10.1001/jamanetworkopen.2023.17952 (PMC10261997; doi:10.1001/jamanetworkopen.2023.17952)
Supplement: Supplement 1. — eTable 1. Walkable Destinations and Birth Weight Outcomes eTable 2. GWG Mediation Analysis for Unhealthy Neighborhood Food Environment and LGA [file jamanetwopen-e2317952-s001.pdf]

Supplemental Online Content

Kinsey EW, Widen EM, Quinn JW, et al. Neighborhood food environment and birth weight outcomes in New York City. *JAMA Netw Open.* 2023;6(6):e2317952. doi:10.1001/jamanetworkopen.2023.17952

eTable 1. Walkable Destinations and Birth Weight Outcomes

eTable 2. GWG Mediation Analysis for Unhealthy Neighborhood Food Environment and LGA

This supplemental material has been provided by the authors to give readers additional information about their work.

© 2023 Kinsey EW et al. *JAMA Network Open.*

eTable 1. Walkable Destinations and Birth Weight Outcomes

	Unadjusted n=97,268	Adjusted[a] n=96,975	Adjusted[b] n=96,961	Sensitivity Analysis Restricted to Full Term Births[b] n=90,469
	Risk Ratio (95% CI)			
Small-for-gestational age				
Poverty rate quartile				
1	ref.	ref.	ref.	ref.
2	0.94 (0.87-1.03)	0.93 (0.86-1.00)	0.93 (0.86-1.00)	0.92 (0.85-0.99)
3	1.02 (0.95-1.10)	0.99 (0.92-1.06)	0.98 (0.92-1.05)	0.98 (0.91-1.05)
4	1.05 (0.97-1.14)	1.02 (0.94-1.20)	1.01 (0.93-1.09)	1.01 (0.93-1.09)
Walkable destinations density quartile				
1	ref.	ref.	ref.	ref.
2	1.09 (0.99-1.19)	1.07 (0.98-1.16)	1.07 (0.98-1.16)	1.08 (1.00-1.16)
3	1.10 (0.98-1.22)	1.09 (1.00-1.20)	1.08 (0.99-1.18)	1.09 (1.00-1.18)
4	1.06 (0.95-1.18)	1.05 (0.96-1.14)	1.03 (0.95-1.12)	1.04 (0.96-1.13)
Large-for-gestational age				
	n=92,467	n=92,200	n=92,185	n=85,865
Poverty rate quartile				
1	ref.	ref.	ref.	ref.
2	1.00 (0.93-1.09)	1.01 (0.94-1.08)	1.01 (0.94-1.08)	1.02 (0.96-1.09)
3	1.03 (0.94-1.12)	1.02 (0.95-1.11)	1.02 (0.95-1.10)	1.03 (0.96-1.11)
4	0.99 (0.90-1.08)	0.97 (0.90-1.05)	0.98 (0.91-1.05)	0.98 (0.91-1.05)
Walkable destinations density quartile				
1	ref.	ref.	ref.	ref.
2	0.98 (0.90-1.07)	1.02 (0.95-1.10)	1.05 (0.98-1.13)	1.04 (0.97-1.12)
3	0.96 (0.88-1.05)	1.00 (0.94-1.08)	1.05 (0.98-1.13)	1.04 (0.96-1.12)
4	0.95 (0.86-1.05)	1.02 (0.94-1.10)	1.08 (0.99-1.16)	1.08 (1.00-1.18)

[a] Adjusted for maternal age, race and ethnicity, nativity, educational level, smoking status, parity, child sex, insurance type, and pre-pregnancy body mass index (continuous)
[b] Adjusted for maternal age, race and ethnicity, nativity, educational level, smoking status, parity, child sex, insurance type, pre-pregnancy body mass index (continuous), and GWG z-score

© 2023 Kinsey EW et al. *JAMA Network Open.*

eTable 2. GWG Mediation Analysis for Unhealthy Neighborhood Food Environment and LGA

	Direct Effect	Indirect Effect	Total Effect
	Risk Ratio (95% CI)		
Large-for-gestational age			
Unhealthy Food Density			
Quartile 1 vs quartile 2	1.16 (1.02-1.31)	0.98 (0.97-0.99)	1.13 (1.00-1.28)
Quartile 1 vs quartile 3	1.17 (1.00-1.36)	0.97 (0.95-0.98)	1.13 (0.97-1.32)
Quartile 1 vs quartile 4	1.18 (0.99-1.41)	0.94 (0.93-0.96)	1.11 (0.93-1.33)

Adjusted for maternal age, race and ethnicity, nativity, educational level, smoking status, parity, child sex, insurance type, and pre-pregnancy body mass index (continuous)

We used the Stata macro from Vanderweele to conduct mediation analysis using the "paramed" command with a poisson outcome regression and a linear mediator regression. As the code does not allow for a categorical exposure variable, we performed three separate analyses with unhealthy food density as a dichotomous exposure (1st vs 2nd, 1st vs 3rd and 1st vs 4th quartiles). The models were controlled for the same covariates as in our main outcome models and categorical covariates (race/ethnicity, education, parity and insurance type) were coded as a series of indicator variables.

© 2023 Kinsey EW et al. *JAMA Network Open.*
