# Supplementary material for: Neighborhood Food Environment and Birth Weight Outcomes in New York City
Source: JAMA Netw Open. 2023 Jun 12;6(6):e2317952. doi: 10.1001/jamanetworkopen.2023.17952 (PMC10261997; doi:10.1001/jamanetworkopen.2023.17952)
Supplement: Supplement 2. — Data Sharing Statement [file jamanetwopen-e2317952-s002.pdf]

Data Sharing Statement

Kinsey. Neighborhood Food Environment and Birth Weight Outcomes in New York City. *JAMA Netw Open*. Published June 12, 2023. doi:10.1001/jamanetworkopen.2023.17952

Data
Data available: No

Additional Information
Explanation for why data not available: The Vital Statistics birth certificate data used in this study are collected and maintained by the New York City Department of Health and Mental Hygiene.
